# Epigenetic Mechanisms and Microbiota as a Toolbox for Plant Phenotypic Adjustment to Environment

**DOI:** 10.3389/fpls.2015.01159

**Published:** 2015-12-23

**Authors:** Nathan Vannier, Cendrine Mony, Anne-Kristel Bittebière, Philippe Vandenkoornhuyse

**Affiliations:** ^1^Université de Rennes 1, CNRS, UMR6553 EcoBioRennes, France; ^2^Université de Lyon 1, CNRS, UMR5023 LEHNAVilleurbanne, France

**Keywords:** plant plasticity, phenotypic plasticity, microbiota, epigenetics, rapid adaptation

## Abstract

The classic understanding of organisms focuses on genes as the main source of species evolution and diversification. The recent concept of genetic accommodation questions this gene centric view by emphasizing the importance of phenotypic plasticity on evolutionary trajectories. Recent discoveries on epigenetics and symbiotic microbiota demonstrated their deep impact on plant survival, adaptation and evolution thus suggesting a novel comprehension of the plant phenotype. In addition, interplays between these two phenomena controlling plant plasticity can be suggested. Because epigenetic and plant-associated (micro-) organisms are both key sources of phenotypic variation allowing environmental adjustments, we argue that they must be considered in terms of evolution. This ‘non-conventional’ set of mediators of phenotypic variation can be seen as a toolbox for plant adaptation to environment over short, medium and long time-scales.

Evolution is driven by selection forces acting on variation among individuals. Understanding the sources of such variation that has led to the diversification of living organisms, is therefore of major importance in evolutionary biology. Diversification is largely thought to be controlled by genetically based changes induced by ecological factors (Schluter, 1994, 2000). Phenotypic plasticity, i.e., the ability of a genotype to produce different phenotypes (Bradshaw, 1965; Schlichting, 1986; Pigliucci, 2005), is a key developmental parameter for many organisms and is now considered as a source of adjustment and adaptation to biotic and abiotic constraints (e.g., West-Eberhard, 2005; Anderson et al., 2011). However, many current studies still focus on genetically generated plasticity to predict and model biodiversity response to a changing climate (Peck et al., 2015), omitting considerable individual variability. In addition, it is striking how poorly the variability is integrated and that both experiments and models most often measure population averages (Peck et al., 2015).

Because of their sessile lifestyle, plants are forced to cope with local environmental conditions and their survival subsequently relies greatly on plasticity (Sultan, 2000). Plastic responses may include modifications in morphology, physiology, behavior, growth or life history traits (Sultan, 2000). In this context, the developmental genetic pathways supporting plasticity allow a rapid response to environmental conditions (Martin and Pfennig, 2010) and the genes underlying these induced phenotypes are subjected to selection (Pfennig et al., 2010). If selection acts primarily on phenotype, the environmental constraints an organism has to face can lead either to directional selection or disruptive selection of new phenotypes (Pfennig et al., 2010). Thus, novel traits can result from environmental induction followed by genetic accommodation of the changes (West-Eberhard, 2005). These accommodated novelties, because they are acting in response to the environment, are proposed to have greater evolutionary impact than mutation-induced novelties (West-Eberhard, 2005). The links between genotype and phenotypes are often blurred by factors including (i) epigenetic effects inducing modifications of gene expression, post-transcriptional and post-translational modifications, which allow a quick response to an environmental stress (Shaw and Etterson, 2012) and (ii) the plant symbiotic microbiota recruited to dynamically adjust to environmental constraints (Vandenkoornhuyse et al., 2015). We investigate current knowledge regarding the evolutionary impact of epigenetic mechanisms and symbiotic microbiota and call into question the suitability of the current gene-centric view in the description of plant evolution. We also address the possible interactions between the responsive epigenetic mechanisms and symbiotic interactions shaping the biotic environment and phenotypic variations.

## Genotype-Phenotype Link: Still Appropriate?

In the neo-Darwinian synthesis of evolution (Mayr and Provine, 1998), phenotypes are determined by genes. The underlying paradigm is that phenotype is a consequence of genotype (Alberch, 1991) in a non-linear interaction due to overdominance, epistasis, pleiotropy, and covariance of genes (see Alberch, 1991; Pigliucci, 2005). Both genotypic variations and the induction of phenotypic variation through environmental changes have been empirically demonstrated, thus highlighting the part played by the environment in explaining phenotypes. These phenotypes are consequences of the perception, transduction and integration of environmental signals. The latter is dependent on environmental parameters, including (i) the reliability or relevance of the environmental signals (Huber and Hutchings, 1997), (ii) the intensity of the environmental signal which determines the response strength (Hodge, 2004), (iii) the habitat patchiness (Alpert and Simms, 2002) and (iv) the predictability of future environmental conditions with current environmental signals information (Reed et al., 2010). The integration of all these characteristics of the environmental stimulus regulates the triggering and outcomes of the plastic response (e.g., Alpert and Simms, 2002). In this line, recent works have shown that plant phenotypic plasticity is in fact determined by the interaction between plant genotype and the environment rather than by genotype alone (El-Soda et al., 2014). Substantial variations in molecular content and phenotypic characteristics have been repeatedly observed in isogenic cells (Kaern et al., 2005). Moreover, recent analyses of massive datasets on genotypic polymorphism and phenotype often struggle to identify single genetic loci that control phenotypic trait variation (Anderson et al., 2011). The production of multiple phenotypes is not limited to the genomic information and the idea of a genotype–phenotype link no longer seems fully appropriate in the light of these findings. Besides, evidence has demonstrated that phenotypic variations are related to genes-transcription and RNAs-translation, which are often linked to epigenetic mechanisms, as discussed in the following paragraph (Rapp and Wendel, 2005).

## Epigenetics as a Fundamental Mechanism for Plant Phenotypic Plasticity

“Epigenetics” often refers to a suite of interacting molecular mechanisms that alter gene expression and function without changing the DNA sequence (Richards, 2006; Holeski et al., 2012). The best-known epigenetic mechanisms involve DNA methylation, histone modifications and histone variants, and small RNAs. These epigenetic mechanisms lead to enhanced or reduced gene transcription and RNA-translation (e.g., Richards, 2006; Holeski et al., 2012). A more restricted definition applied in this paper considers as epigenetic the states of the epigenome regarding epigenetic marks that affect gene expression: DNA methylation, histone modifications (i.e., histone amino-terminal modifications that act on affinities for chromatin-associated proteins) and histone variants (i.e., structure and functioning), and small RNAs. These epigenetic marks may act separately or concomitantly, and can be heritable and reversible (e.g., Molinier et al., 2006; Richards, 2011; Bilichak, 2012). The induction of defense pathways and metabolite synthesis against biotic and abiotic constraints by epigenetic marks has been demonstrated during the last decade mainly in the model plant species *Arabidopsis* and tomato (e.g., Rasmann et al., 2012; Slaughter et al., 2012; Sahu et al., 2013). Epigenetics is now regarded as a substantial source of phenotypic variations (Manning et al., 2006; Crews et al., 2007; Kucharski et al., 2008; Bilichak, 2012; Zhang et al., 2013) in response to environmental conditions. More importantly, studies have suggested the existence of epigenetic variation that does not rely on genetic variation for its formation and maintenance (Richards, 2006; Vaughn et al., 2007). However, to date, only a few studies have demonstrated the existence of pure natural epi-alleles (Cubas et al., 1999) although they are assumed to play an important role in relevant trait variation of cultivated plants (Quadrana et al., 2014). Similarly to the results observed in mangrove plants (Lira-Medeiros et al., 2010), a recent work on *Pinus pinea* which exhibits high phenotypic plasticity associated with low genetic diversity, discriminated both population and individuals based on cytosine methylation, while the genetic profiles failed to explain the observed phenotype variations (Sáez-Laguna et al., 2014). Epigenetics can provide phenotypic variation in response to environmental conditions without individual genetic diversity. It could hence provide an alternative way or an accelerated pathway for adaptive ‘evolutionary’ changes (Bossdorf et al., 2008). Epigenetic marks could also ‘tag’ a site for mutation: it is known that methylated cytosine is more mutable increasing the opportunity for random mutation to act at epigenetically modified sites.

## Epi-Alleles, Genetic Accommodation and Adaptation

Even if totally independent epigenetic variations (i.e., pure epi-alleles) are scarce and still need to be investigated, the evolutionary significance of the resulting epigenetically induced phenotypic variations is being increasingly debated (Schlichting and Wund, 2014). Assuming that selection acts on phenotypes and that these phenotypes are not always genetically controlled, it can be argued that new phenotypes arising from adaptive plasticity are not *random variants* (West-Eberhard, 2005). Changes in the trait frequency then correspond to a ‘genetic accommodation’ process (West-Eberhard, 2005; Schlichting and Wund, 2014) through which an environmentally induced trait variation becomes genetically determined by a change in genes frequency that affects the trait ‘reaction norm’ (West-Eberhard, 2005; Crispo, 2007). It may also be suggested that genetic accommodation can result from the selection of genetic changes optimizing the novel variant’s adaptive value through modifications in the form, regulation or phenotypic integration of the trait.

In the “adaptation loop,” the effect of environment on plant performance induces the selection of the most efficient phenotype. The epigenetic processes are not the only engines of plant phenotypic plasticity adjustment. Indeed, plants also maintain symbiotic interactions with microorganisms to produce phenotypic variations.

## Plant Phenotypic Plasticity and Symbiotic Microbiota

Plants harbor an extreme diversity of symbionts including fungi (Vandenkoornhuyse et al., 2002) and bacteria (Bulgarelli et al., 2012; Lundberg et al., 2012). During the last decade, substantial research efforts have documented the range of phenotypic variations allowed by symbionts. Examples of mutualist-induced changes in plant functional traits have been reported (Streitwolf-Engel et al., 1997, 2001; Wagner et al., 2014), which modify the plant’s ability to acquire resources, reproduce, and resist biotic and abiotic constraints. The detailed pathways linking environmental signals to this mutualist-induced plasticity have been identified in some cases. For instance, Boller and Felix (2009) highlighted several mutualist-induced signaling pathways allowing a plastic response of plants to virus, pests and pathogens initiated by flagellin/FLS2 and EF-Tu/EFR recognition receptors. Mutualist-induced plastic changes may affect plant fitness by modifying plant response to its environment including (i) plant-resistance to salinity (Lopez et al., 2008), drought (Rodriguez et al., 2008), heat (Redman et al., 2002) and (ii) plant nutrition (e.g., Smith et al., 2009). These additive ecological functions supplied by plant mutualists extend the plant’s adaptation ability (e.g., Vandenkoornhuyse et al., 2015), leading to fitness benefits for the host in highly variable environments (Conrath et al., 2006) and therefore can affect evolutionary trajectories (e.g., Brundrett, 2002). In fact, mutualism is a particular case of symbiosis (i.e., long lasting interaction) and is supposed to be unstable in terms of evolution because a mutualist symbiont is expected to improve its fitness by investing less in the interaction. Reciprocally, to improve its fitness a host would provide fewer nutrients to its symbiont. Thus, from a theoretical point of view, a continuum from parasite to mutualists is expected in symbioses. However, the ability of plants to promote the best cooperators by a preferential C flux has been demonstrated both in *Rhizobium*/ and Arbuscular Mycorrhiza/*Medicago truncatula* interactions (Kiers et al., 2007, 2011). Thus, the plant may play an active role in the process of mutualist-induced environment adaptation as it may be able to recruit microorganisms from soil (for review Vandenkoornhuyse et al., 2015) and preferentially promote the best cooperators through a nutrient embargo toward less beneficial microbes (Kiers et al., 2011). In parallel, vertical transmission or environmental inheritance of a core microbiota is suggested (Wilkinson and Sherratt, 2001) constituting a “continuity of partnership” (Zilber-Rosenberg and Rosenberg, 2008). Thus the impact on phenotype is not limited to the individual’s lifetime but is also extended to reproductive strategies and to the next generation. Indeed, multiple cases of alteration in reproductive strategies mediated by mutualists such as arbuscular mycorrhizal fungi (Sudová, 2009) or endophytic fungi (Afkhami and Rudgers, 2008) have been reported. Such microbiota, being selected by the plant and persisting through generations, may therefore influence the plant phenotype and be considered as a powerhouse allowing rapid buffering of environmental changes (Vandenkoornhuyse et al., 2015). The idea of a plant as an independent entity on the one hand and its associated microorganisms on the other hand has therefore recently matured toward understanding the plant as a holobiont or integrated “super-organism” (e.g., Vandenkoornhuyse et al., 2015).

## Holobiont Plasticity and Evolution

If the holobiont can be considered as the unit of selection (Zilber-Rosenberg and Rosenberg, 2008), even though this idea is still debated (e.g., Leggat et al., 2007; Rosenberg et al., 2007), then the occurrence of phenotypic variation is enhanced by the versatility of the holobiont composition, both in terms of genetic diversity (i.e., through microbiota genes mainly) and phenotypic changes (induced by mutualists). Different mechanisms allowing a rapid response of the holobiont to these changes have been identified (1) horizontal gene transfer between members of the holobiont (i.e., transfer of genetic material between bacteria; Dinsdale et al., 2008) (2) microbial amplification (i.e., variation of microbes abundance in relation to environment variation) and (3) recruitment of new mutualists within the holobiont (Vandenkoornhuyse et al., 2015). In this model, genetic novelties in the hologenome (i.e., the combined genomes of the plant and its microbiota, the latter supporting more genes than the host) are a consequence of interactions between the plant and its microbiota. The process of genetic accommodation described in Section “Epi-Alleles, Genetic Accommodation and Adaptation,” impacts not only the plant genome but can also be expanded to all components of the holobiome and may thus be enhanced by the genetic variability of microbiota. In the holobiont, phenotypic plasticity is produced at different integration levels (i.e., organism, super-organism) and is also genetically accommodated or assimilated at those scales (i.e., within the plant and mutualist genomes and therefore the hologenome). The holobiont thus displays greater potential phenotypic plasticity and a higher genetic potential for mutation than the plant alone, thereby supporting selection and the accommodation process in the hologenome. In this context, the variability of both mutualist-induced and epigenetically induced plasticity in the holobiont could function as a “toolbox” for plant adaptation through genetic accommodation. Consequently, mechanisms such as epigenetics allowing a production of phenotypic variants in response to the environment should be of importance in the holobiont context.

## Do Microbiota and Epigenetic Mechanisms Act Separately or can they Interact?

**FIGURE 1:**
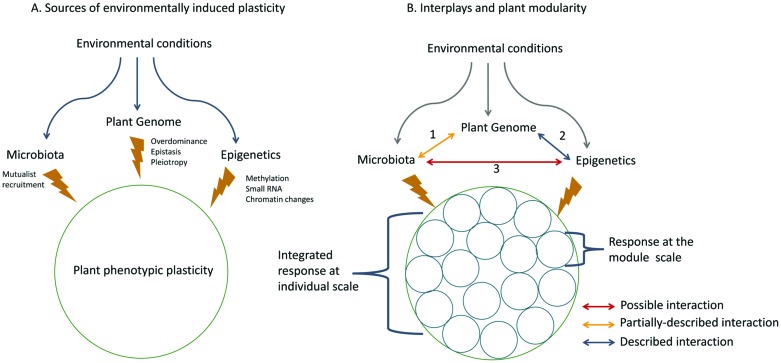
**(A)** Plant phenotypic plasticity is triggered by environmental constraints. Phenotypic changes induced are not solely genetically controlled but are also based on either epigenetic marks or plant microbiota by recruitment of mutualists. This plant ‘toolbox’ allows a rapid response to environmental constraints. **(B)** The control over plant phenotypic plasticity may cross-talk or synergistically interplay with different possible interactions. (1) Co-evolution plant-symbiont (2) Interplay genetic/epigenetics (3) Interaction between epigenetics and microbiota. These mechanisms also act at the modular scale of plant structure.

Both epigenetic and microbiota interactions allow plants to rapidly adjust to environmental conditions and subsequently support their fitness (**Figure 1**). Phenotypic changes ascribable to mutualists and mutualists transmission to progeny are often viewed as epigenetic variation (e.g., Gilbert et al., 2010). However, this kind of plasticity is closer to an “interspecies induction of changes” mediated by epigenetics rather than “epigenetics-induced changes” based solely on epigenetic heritable mechanisms (see section on epigenetics for a restricted definition). Apart from the difficulty of drawing a clear line between epigenesis and epigenetics (Jablonka and Lamb, 2002), evidence is emerging of the involvement of epigenetic mechanisms in mutualistic interactions. An experiment revealed changes in DNA adenine methylation patterns during the establishment of symbiosis (Ichida et al., 2007), suggesting an effect of this interaction on the bacterial epigenome or at least, a role of epigenetic mechanisms in symbiosis development. Correct methylation status seems also to be required for efficient nodulation in the *Lotus japonicus – Mesorhizobium loti* symbiosis (Ichida et al., 2009) and miRNA “miR-397” was only induced in mature nitrogen-fixing nodules (De Luis et al., 2012). As epigenetic mechanisms are involved in the development of symbiosis, we assume that epigenetic phenomena may have significant effects on mutualist associations. As yet, little is known about the epigenetic effects and responses underlying host–symbiont interactions. These epigenetic mechanisms and microbiota sources of plant phenotypic plasticity may act synergistically although this idea has never convincingly been addressed. As far as we know, different important issues bridging epigenetic mechanisms and microbiota remain to be elucidated such as (1) the frequency of epigenetic marking in organisms involved in mutualistic interactions, (2) the range of phenotypic plasticity associated with these marks either in the plant or in microorganisms, (3) the consequences of these marks for holobiont phenotypic integration, (4) the functional interplay between epigenetic mechanisms and microbiota in plant phenotype expression, (5) the inheritance of epigenetic mechanisms and thus their impact on symbiosis development, maintenance and co-evolution. To answer these questions, future studies will need to involve surveys of plant genome epigenetic states (e.g., methylome) in response to the presence/absence of symbiotic microorganisms. Recent progress made on bacteria methylome survey methods should represent useful tools to design future experiment on this topic (Sánchez-Romero et al., 2015).

Although research on the interaction between microbiota and epigenetics is in its infancy in plants, recent works mostly on humans support existing linkages. Indeed, a clear link has been evidenced between microbiota and human behavior (Dinan et al., 2015). Other examples of microbiota effects are their (i) deep physiological impact on the host through serotonin modulation (Yano et al., 2015) and (ii) incidence on adaptation and evolution of the immune system (Lee and Mazmanian, 2010). Such findings should echo in plant-symbionts research and encourage further investigations on this topic.

More broadly, and despite the above-mentioned knowledge gaps, our current understanding of both epigenetic mechanisms and the impact of microbiota on the expression of plant phenotype, invites us to take those phenomena into consideration in species evolution and diversification.

## ‘Extended Phenotype’ and ‘Hologenome Theory’

**FIGURE 2:**
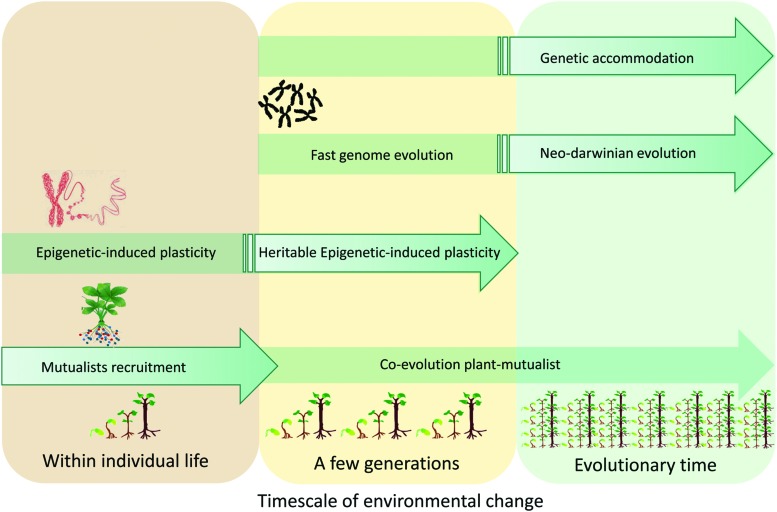
**A plant’s phenotypic variations can be inherited even in the case of a phenotypic trait not controlled by a gene/genome variation.** This rapid response to environmental change involves epigenetic mechanisms and/or microorganisms recruitment within the plant microbiota. Heritable transgenerational plasticity mediated by epigenetic mechanisms and/or mutualists could be followed by genetic accommodation and long term adaptation.

Microbiota and epigenetic mechanisms play different but complementary roles in producing phenotypic variations which are then subjected to selective pressure. Diversification of traits is suggested to depend on evolutionary time (necessary for the accumulation of genetic changes, i.e., Martin and Pfennig, 2010) but rapid shifts in plant traits, as allowed by both microbiota and epigenetics, would provide accelerated pathways for their evolutionary divergence. In addition, such rapid trait shifts also permit rapid character displacement. Induction of DNA methylation may occur more rapidly than genetic modifications and could therefore represent a way to cope with environmental constraints on very short time scales (during the individual’s lifetime; Rando and Verstrepen, 2007). In parallel, microbiota-induced plasticity is achieved both at a short time scale (i.e., through recruitment) and at larger time scales (i.e., through symbiosis evolution; **Figure 2**). Because of the observation of transgenerational epigenetic inheritance, the relevance of epigenetically induced variations is a current hot topic in the contexts of evolutionary ecology and environmental changes (Bossdorf et al., 2008; Slatkin, 2009; Zhang et al., 2013; Schlichting and Wund, 2014). This has stimulated renewed interest in the ‘extended phenotype’ (Dawkins, 1982). The central idea of Dawkins ‘extended phenotype’ (Dawkins, 1982) is that phenotype cannot be limited to biological processes related to gene/genome functioning but should be ‘extended’ to consider all effects that a gene/genome (including organisms behavior) has on its environment. For example, the extended phenotype invites us to consider not only the effect of the plant genome on its resources acquisition but also the effect of the genome on the plant symbionts as well as on nutrient availability for competing organisms. More recently the development of the ‘hologenome theory’ (Zilber-Rosenberg and Rosenberg, 2008) posits that evolution acts on composite organisms (i.e., host and its microbiome) with the microbiota being fundamental for their host fitness by buffering environmental constraints. Both the ‘extended phenotype’ concept and ‘hologenome theory’ admit that the environment can leave a “footprint” on the transmission of induced characters. Thus, opportunities exist to revisit our understanding of plant evolution to embrace both environmentally induced changes and related ‘genetic accommodation’ processes.

## Conflict of Interest Statement

The authors declare that the research was conducted in the absence of any commercial or financial relationships that could be construed as a potential conflict of interest. The reviewer Stijn Spaepen and handling Editor Stéphane Hacquard declared their shared affiliation, and the handling Editor states that, nevertheless, the process met the standards of a fair and objective review.
